# Sun Exposure and Asthma Prevalence in Spanish Schoolchildren and Adolescents in the Global Asthma Network (GAN) Phase I Study: A Semi-Individual Cross-Sectional Study

**DOI:** 10.3390/jcm15166238

**Published:** 2026-08-12

**Authors:** Alberto Arnedo-Pena, Saeed Fattahi, Inés Aguinaga-Ontoso, Alberto Bercedo-Sanz, Carlos González-Díaz, Angel López-Silvarrey Varela, Antonia Elena Martínez-Torres, Javier Pellegrini-Belinchón, Manuel Sánchez-Solís, Luis García-Marcos, the Spanish GAN Group

**Affiliations:** 1 Centro de Salud Pública, 12003 Castelló de la Plana, Spain; albertoarnedopena@gmail.com; 2 CIBER de Epidemiologia y Salud Pública (CIBERESP), 28029 Madrid, Spain; ines.aguinaga@unavarra.es; 3 Departamento de Ciencias de la Salud, Universidad Pública de Navarra, 31006 Pamplona, Spain; 4 Área de Pediatría, Facultad de Medicina, Universidad de Murcia, 30071 Murcia, Spain; fattahisaeed957@gmail.com; 5 Grupo de Epidemiología Clínica, Área de Epidemiología y Salud Pública, Instituto de Investigación Sanitaria de Navarra (IdiSNA), 31008 Pamplona, Spain; 6 Centro de Salud Los Castros, Servicio Cántabro de Salud, 39011 Santander, Spain; drbercedo@gmail.com; 7 Instituto de Investigación Sanitaria Valdecilla (IDIVAL), 39011 Santander, Spain; 8 Unidad de Alergia Infantil, Hospital Universitario Basurto, 48013 Bilbao, Spain; manuelcarlos.gonzalezdiaz@osakidetza.eus; 9 Fundación María José Jove, 15008 A Coruña, Spain; angel.lopez-silvarrey.varela@sergas.es; 10 Servicio Galego de Saúde (SERGAS), 15006 La Coruña, Spain; 11 Grupo de Investigación en Enfermería, Unidad de Neumología y Alergia Pediátrica, Hospital Infantil Universitario Virgen de la Arrixaca, Instituto Murciano de Investigación Biosanitaria IMIB, 30120 Murcia, Spain; elenamt@um.es; 12 Centro de Salud Pizarrales, 37006 Salamanca, Spain; jpellegrini@usal.es; 13 Departamento de Ciencias Biomédicas y del Diagnóstico, Universidad de Salamanca, 37008 Salamanca, Spain; 14 Unidad de Neumología y Alergia Pediátrica, Hospital Infantil Universitario Virgen de la Arrixaca, Universidad de Murcia, Instituto Murciano de Investigación Biosanitaria IMIB, 30120 Murcia, Spain; msolis@um.es

**Keywords:** asthma, prevalence, schoolchildren, adolescents, sunshine hours, global solar surface irradiance, multilevel logistic regression

## Abstract

**Background/Objective:** In the multifactorial etiology of asthma, environmental factors, including sun exposure, measured either by sunshine hours (SHs) or global solar surface irradiance (GSSI), are critical. The objective of this study is to estimate the association between sun exposure and the prevalence of asthma in Spanish schoolchildren (6–7 years old) and adolescents (13–14 years old) from six geographic centers in Spain (A Coruña, Bilbao, Cantabria, Cartagena, Pamplona and Salamanca) included in the Global Asthma Network (GAN) study during 2016–2019. **Methods**: Measurements of SHs and GSSI were obtained from the Spanish “Agencia Estatal de Meteorología”. A semi-individual cross-sectional design was used, and multilevel logistic regression models were employed, with adjustments for age, sex, temperature, relative humidity and gross domestic product, taking the center as the reference level. Permutation tests were performed to account for the low number of centers. **Results:** Increases in both SHs and GSSI were associated with a lower prevalence of “asthma ever” in the 6–7 and 13–14 years age groups. An increase of 100 annual sunshine hours was associated with a lower prevalence of “asthma ever” among schoolchildren (adjusted odds ratio [aOR] = 0.82; 95% confidence interval [CI], 0.79–0.85); from the crude model, permutation *p* = 0.08. In adolescents, the corresponding estimates were aOR = 0.90 (95% CI, 0.87–0.94); from the crude model, permutation *p* = 0.02. An increase of 1 kWh m^−2^ day^−1^ in GSSI was associated with a lower prevalence of “asthma ever” among schoolchildren (aOR = 0.15; 95% CI, 0.10–0.23); from the crude model, permutation *p* = 0.25. In adolescents, the corresponding estimates were aOR = 0.37 (95% CI, 0.25–0.54); from the crude model, permutation *p* = 0.01. **Conclusions:** The results of this study suggest that sun exposure, measured either as SHs or GSSI, may be protective against asthma in adolescents. In contrast, the association between sun exposure and the prevalence of “asthma ever” in schoolchildren was not significant, although it followed a similar trend. Confirmation of these findings in other geographical settings could improve our understanding of the role of sun exposure in asthma prevention and control.

## 1. Introduction

According to asthma’s multifactorial etiology, some effects of climate on its prevalence have already been proven; studied meteorological variables include temperature, humidity, and thunderstorms [1,2,3,4,5].

More recently, it has been hypothesized that vitamin D deficiency may be associated with the increase in childhood asthma in Western countries [6,7]. This hypothesis has led to some observational studies on the prevalence of asthma and its association with sun exposure, in which geographical latitude or sunshine hours per year were used as proxies of vitamin D exposure [8,9]. These studies found an inverse relationship between childhood asthma prevalence and sun exposure in different countries, supporting this hypothesis.

However, the effects of vitamin D on asthma incidence or prevalence continue to be disputed, and there is no consensus on the mechanisms, factors, or associated comorbidities [10,11,12]. Prenatal supplementation of vitamin D has been found to have no significant, or very limited, effect on asthma and allergies in children [13,14], despite some clinical trials observing significant decreases [15,16]. On the other hand, it was found that vitamin D supplementation in infants did not result in a reduction in the prevalence of wheezing or asthma after several years of follow-up [17,18,19]. Additionally, no effect on asthma control has been observed [20], and in a Mendelian randomization study, the level of vitamin D was not associated with allergic asthma [21]. However, vitamin D concentration was associated with decreased asthma incidence in a prospective cohort of adults in China [22], and an ecological study in England showed that in areas with the highest ultraviolet radiation, weighted by the pre-vitamin D action spectrum, there was a reduced prevalence of asthma in adults [23].

Epidemiological studies on the prevalence of asthma and geographical asthma variations have seen renewed interest in the context of climate change [24], with studies measuring the effect of sun exposure in new populations and distinguishing between sunshine hours (SHs) (a measure of the time of solar radiation) and global solar surface irradiance (GSSI) (total energy received from the sun). Sun exposure can cause diseases, such as skin cancer, and dermatological damage, but can also have health benefits [25,26]. Asthma-related benefits include increased vitamin D production, decreased transmission of infectious diseases, moderation of pollen seasons, and positive immunomodulation [27,28].

A worldwide study called the Global Asthma Network (GAN) study [29] measured the prevalence of asthma, eczema and allergic rhinitis in schoolchildren and adolescents in many countries, following the methods of the International Study of Asthma and Allergies in Childhood (ISAAC) established between 1994 and 2004 [30]. In Spain, five centers fully participated in this study from 2016 to 2019: Bilbao, Cantabria, and A Coruña on the Atlantic coast; Salamanca in the central east; and Cartagena on the Mediterranean coast. Pamplona, in the interior north, was the site of a survey during the same period, but this only included partial application of the GAN questionnaire and thus was not incorporated in the global dataset.

The GAN study provides an opportunity to better study the effect of sun exposure on the prevalence of asthma in different geographical areas, expanding the ISAAC results obtained in 2011 [9]. The objective of the present study was to estimate the association between sun exposure, measured in sunshine hours (SHs) or global solar surface irradiation (GSSI), and the prevalence of asthma in schoolchildren and adolescents in Spain.

## 2. Materials and Methods

### 2.1. Study Design and Variable Definitions

The study has a semi-individual design [31]. This design has been used in studies on environmental pollution [32] and has advantages over an ecological design, as outcomes are measured at the individual level and predictors are measured as aggregate data. Adjustment for aggregated variables can reduce ecological bias.

The dependent variable was “asthma ever” based on the questionnaire used in the GAN study. In children aged 6–7 years old, “asthma ever” was defined as a positive answer (from parents) to the question “Has this child ever had asthma? (Yes/No)”, while in adolescents, the question “Have you ever had asthma? (Yes/No)” was answered by the adolescents themselves. For validation purposes, we also performed a sensitivity analysis to explore the consistency between the question on “asthma ever” and an “asthma diagnosis”. This was defined as a positive answer to the question “Was this child’s asthma confirmed by a doctor? (Yes/No)”, from the parents of schoolchildren, and “Was asthma confirmed by a doctor? (Yes/No)”, from adolescents themselves. The data on the prevalence of asthma were retrieved from the studies of the Spanish Group of the Global Asthma Network from 2016 to 2019 [33,34].

SHs and GSSI were used as predictor variables. SHs (from 1981 to 2010) were obtained from the Spanish “Agencia Estatal de Meteorología (AEMET)”, and the daily mean GSSI (period: 1983–2005) for the capital cities of each center’s province was acquired via satellite measurement [35]. Other variables included as potential confounding factors were the age and sex of the participant; annual average of temperature and relative humidity (period: 1981–2010), also taken from the AEMET [36]; and the annual averages of gross domestic product (GDP) per capita of the province where each center is located (2016–2019), obtained from the Spanish “Instituto Nacional de Estadística (INE)” [37].

### 2.2. Statistical Analysis

Asthma prevalence was calculated by dividing the number of positive answers to the aforementioned questions by the total number of participants for the two age groups for each center, expressed as percentages. Logistic regression was used to compare the asthma prevalence rates of each center using crude odds ratios (cORs) with 95% confidence intervals (CIs). Temperature was measured in degrees centigrade, relative humidity was taken as the percentage of water saturation in the air, and GDP was measured in Euros (€). Annual sunshine hours were divided by 100 to facilitate interpretation of the results, and associations between asthma prevalence (“asthma ever” and physician-diagnosed asthma) and sun exposure (SHs and GSSI) in schoolchildren and adolescents were estimated using multilevel logistic regression models, with odds ratios (ORs) as the measure of association.

Multilevel logistic regression models were used in the multivariable analysis, and the center (Bilbao, Cantabria, A Coruña, Pamplona, Salamanca and Cartagena) was considered the reference level of the six analyzed zones and was also the random effect component. Sun exposure (SHs and GSSI), temperature, relative humidity and GDP were the fixed-effect components. This allows for comparing variations between centers and between participants while controlling for variables at multiple levels [38,39]. Table 1 presents the parameters of multilevel models and the null model, with covariates for “asthma ever” and “asthma diagnosis”, considering SHs and GSSI. It is worth noting that the inter-class correlation coefficient (ICC) of the null model for asthma diagnosis was 7.6%, which explains the effect of the center, with a median odds ratio (MOR) of 1.64, which quantifies the effect of center for schoolchildren; the ICC was 3.41% and MOR was 1.38 for adolescents. For “asthma ever”, these values were 6.9% and 1.60 for schoolchildren and 2.45% and 1.32 for adolescents, respectively. The full models with predictor variables exhibited substantially reduced variances, ICCs, and MORs for both SHs and GSSI. Model comparison via Akaike’s information criterion (AIC), Bayesian information criterion (BIC), log likelihood ratio (LLR), and deviance indicated the full models gave a better fit, as also evidenced by different models’ graphs of predicted and observed values [40].

**Figure 1:**
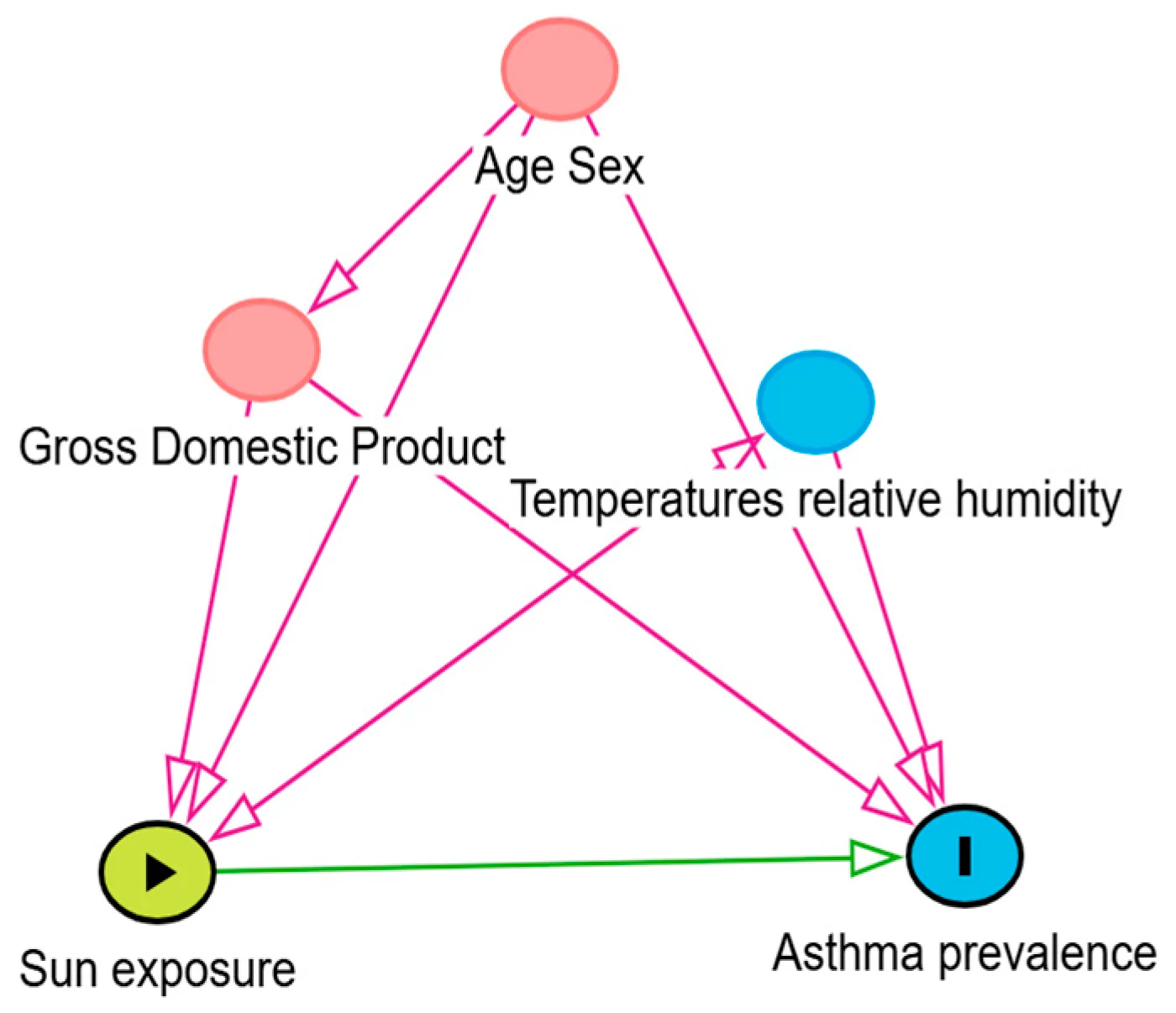
Directed Acyclic Graph (DAG) diagram showing the effect of sun exposure (exposure) on asthma prevalence (outcome). Ancestors of both exposure and outcome are shown in red, and ancestors of the outcome are shown in blue (generated using the DAGitty program, version 3.1).

Crude and adjusted odds ratios (cORs; aORs) with a 95% CI were calculated. These models allowed for adjustment for potential confounding factors such as age, sex, temperature, relative humidity, and GDP. We followed the Directed Acyclic Graph (DAG) method [41] to control for potential confounding factors, using DAGitty software version 3.1 to create a DAG diagram (Figure 1) [42]. Given the small number of centers included in the study, permutation tests were performed for the crude SH and GSSI multilevel models using the “ritest” command developed by S. Heß, with 100 replications [43]. In addition, a leave-one-center-out sensitivity analysis was conducted by sequentially removing one center at a time from the crude SH and GSSI analysis to identify centers that might disproportionately influence the associations between asthma prevalence and sun exposure or the estimated odds ratios (ORs). Statistical analyses were performed using Stata™ version 14.2 (StataCorp LLC, College Station, TX, USA).

### 2.3. Ethics

The GAN study in Spain was approved by the Clinical Research Ethical Committee of the “Hospital Clínico Universitario de la Arrixaca”, Murcia. Passive informed consent was applied to questionnaires, which were anonymized.

## 3. Results

The GAN study took place in Spain in 2016–2019, with a total of 17,215 schoolchildren, aged 6–7 years old, and 19,943 adolescents, aged 13–14 years old, participating. The overall response rate was 62.6% (17,215/27,497) for schoolchildren and 81.3% (19,943/24,516) for adolescents.

Table 2 shows the asthma prevalence for “asthma ever” and “asthma diagnosis” in the six centers for both age groups, and includes a comparison, conducted by means of logistic regression analyses, using the center with the lowest prevalence as the reference. There are large differences between the six centers: the highest prevalence of “asthma ever” and “asthma diagnosis” in schoolchildren was observed in Bilbao, at 22.7% (OR = 4.34; 95% CI 3.59–5.24) and 21.0% (OR = 4.49; 95% CI 3.68–5.47), respectively. In adolescents, the highest prevalence was again in Bilbao at 29.9% (OR= 2.43; 95% CI 2.16–2.74) for “asthma ever” and 26.9% (OR = 2.78; 95% CI 2.45–3.15) for “asthma diagnosis”, followed by Cantabria, at 17.2% (OR = 3.07; 95% CI 2.54–3.73) and 15.1% (OR = 3.01; 95% CI 2.46–3.67) in schoolchildren and 23.6% (OR = 1.76; 95% CI 1.56–1.98) and 20.6% (OR = 1.96; 95% CI 1.73–2.22) in adolescents, respectively. A low asthma prevalence was observed both in schoolchildren and adolescents in A Coruña and Salamanca. Pamplona exhibited the lowest prevalence of “asthma ever” and “asthma diagnosis” in schoolchildren at 6.3% and 5.6%, respectively, while Cartagena had the lowest in adolescents, at 14.9% and 11.7%, respectively.

Table 3 shows SHs, GSSI, temperature (°C), relative humidity (%), and GDP per capita (€) in the six centers. Bilbao and Cantabria had the lowest number of sunshine hours/year and daily solar irradiance, while Salamanca and Cartagena had the highest, and the mean annual temperatures were lowest in Salamanca and Pamplona and highest in Cartagena. Relative humidity exhibited small differences between centers, and per capita GDP was the highest in Pamplona and Bilbao and the lowest in Salamanca and Cartagena.

Table 4 shows the results of the multilevel logistic regression models for “asthma ever”, including SHs and GSSI, in the two age groups and the six centers. In the adjusted models, an increase of 100 sunshine hours per year was associated with a significant decrease in the prevalence of “asthma ever” in schoolchildren (aOR = 0.82; 95% CI 0.79–0.85) and adolescents (aOR = 0.90; 95% CI 0.87–0.94). On the other hand, an increase of one kWh m^−2^ day^−1^ in daily GSSI significantly reduced the prevalence of “asthma ever” in schoolchildren (aOR = 0.15; 95% CI 0.10–0.23) and adolescents (aOR = 0.37; 95% CI 0.25–0.54). Thus, the reducing effect of GSSI on the prevalence of “asthma ever” was higher than that of SHs in both groups.

During the sensitivity analysis (Table 5), the prevalence of “asthma diagnosis” and sun exposure was also analyzed using multilevel logistic regression. In the adjusted models, an increase of 100 h in mean annual SHs was significantly associated with a lower prevalence of asthma diagnoses both in schoolchildren (aOR = 0.81; 95% CI 0.77–0.84) and adolescents (aOR = 0.88; 95% CI 0.85–0.92). Similarly, an increase of one kWh m^−2^ day^−1^ in daily GSSI was also associated with a decrease in the prevalence of asthma diagnoses, again both in schoolchildren (aOR = 0.13; 95% CI 0.08–0.20) and adolescents (aOR = 0.32; 95% CI 0.22–0.47). The effects of SHs were lower than those of GSSI in both groups.

Table 6 presents the results of the permutation tests. Among schoolchildren, neither SHs (*p* = 0.08) nor GSSI (*p* = 0.25) was significantly associated with the prevalence of “asthma ever” or the prevalence of physician-diagnosed asthma SHs (*p* = 0.07 and *p* = 0.13, respectively). In contrast, among adolescents, both SHs (*p* = 0.02) and GSSI (*p* = 0.01) were significantly associated with the prevalence of “asthma ever” and the prevalence of physician-diagnosed asthma (*p* = 0.04 and *p* = 0.02, respectively).

Table 7 shows the results of the leave-one-center-out sensitivity analysis, in which one center was sequentially removed from the crude SH and GSSI analyses. Among schoolchildren, removal of the Bilbao center led to non-significant associations between both SHs and GSSI and the prevalence of “asthma ever”, whereas removal of the Cartagena center led to significant associations. A similar pattern was observed for physician-diagnosed asthma: associations became significant after exclusion of the Cartagena center but were no longer significant after exclusion of the Bilbao center. Considerable variations in the estimated odds ratios (ORs) were observed for GSSI.

Among adolescents, significant associations between both SHs and GSSI and the prevalence of “asthma ever” remained after the exclusion of each individual center. Likewise, significant associations with physician-diagnosed asthma persisted regardless of the center removed, with only minor variations in the estimated ORs.

## 4. Discussion

The present study reveals a significantly lower prevalence of “asthma ever” with higher sun exposure, measured by both SHs and GSSI, among adolescents from six Spanish centers. In addition, the sensitivity analyses showed that the prevalence of physician-diagnosed asthma also decreased with increasing sun exposure in this age group. In contrast, among schoolchildren, neither SHs nor GSSI was significantly associated with the prevalence of “asthma ever” or physician-diagnosed asthma, although the direction of the association was similar to that observed in adolescents.

Multilevel models based on a small number of centers may yield spuriously low *p*-values; permutation tests and leave-one-center-out sensitivity analyses are useful to identify this phenomenon. The lack of significant associations in schoolchildren may be explained by the limited between-center variability in asthma prevalence and the disproportionate influence of two centers on the results. However, the effect of sun exposure may be weaker in schoolchildren than in adolescents, or other age-related risk factors may play a more important role in younger children [44,45].

These results are consistent with those of several epidemiological studies that used different proxies for sun exposure, such as latitude, studied in the USA and Australia [8]; sunshine hours, studied in West Europe in the ISAAC study [46]; and, more recently, ultraviolet radiation weighted by the pre-vitamin-D action spectrum, which was investigated in England [23]. The results of the present study are consistent with those of a study by Arnedo-Pena and co-authors [9] in comparable geographical areas, with decreases in asthma prevalence of 0.6% for the 6–7-year-old group and 1.1% for the 13–14-year-old group following a 100 h increase in mean annual sunshine. As found in the present study, centers located in the coastal area of northern Spain with an Atlantic climate (Bilbao, Cantabria, and A Coruña) had higher asthma prevalence rates and lower sun exposure. Pamplona, with a transitional Atlantic–Mediterranean climate, had the lowest asthma prevalence rates in schoolchildren, and Cartagena, with high sun exposure, had the lowest asthma prevalence in adolescents.

The association between GSSI and asthma prevalence was stronger than that observed for SHs in adolescents, suggesting that the reduction in asthma prevalence associated with greater sun exposure may extend beyond the potential effects of sunlight on vitamin D production [47]. Additional mechanisms may include increased resistance to respiratory infections, protection of the mucosal barrier, and enhancement of immune function through multiple biological pathways [21,48,49].

The immunomodulation effect of GSSI, triggered by ultraviolet radiation, produces anti-inflammatory, anti-allergic, and anti-autoimmunity effects via activation of the innate immune system, which would help to reduce asthma susceptibility [28,44,50,51]. In addition, GSSI decreases the presence of house-dust mites, mold and fungal spores, and other allergens through increasing temperature and reducing humidity. Conversely, low solar irradiation increases the risk of allergic reactions to house-dust mites [52,53]. Additionally, solar irradiation can decrease the transmission of viral agents [54] and reduce food sensitization and eczema in infants [47,55], and exposure during the second trimester of pregnancy has been associated with reduced aeroallergen sensitization in two groups of children aged 6 and 12 years old, while irradiation in the third trimester has been associated with increased sensitization to these allergens [56]. In a recent prospective cohort study performed in Paris [57], solar irradiation during both the prenatal and postnatal periods and higher levels of vitamin D in the prenatal period reduced the risk of sensitization of children aged 8–9 years. Periods of intense solar and geomagnetic activity have been shown to improve asthma control, probably by decreasing airway inflammation, in a cohort of asthmatic children in Boston, further supporting the benefits of solar irradiation [58]. Regarding asthma prevalence in different climates, areas with a sunny climate exhibit lower prevalence compared with humid areas [59,60,61,62]. However, it remains unknown whether the potential effects of vitamin D on asthma depend on the asthma phenotype. Multi-omics studies may help to better characterize this association [63].

The strengths of this study include the following: (1) The high number of participants in each center and (2) a particularly high participation rate in the 13–14-year-old group and a good participation rate in the 6–7-year-old group. (3) The use of the validated asthma symptom questionnaire from the ISAAC and GAN studies [29,34] and (4) the use of the Spanish official meteorology agency-measured sun exposure data, with a satellite-based determination of GSSI. (5) The multilevel logistic regression models allowed us to examine the individual prevalence of asthma while controlling for center characteristics and other potential confounding factors. (6) Finally, the sensitivity analysis considering doctor-confirmed asthma diagnoses strengthened the initial results for “asthma ever”.

However, this study has some limitations: Firstly, the semi-individual design reduces the ecological fallacy, but this cannot be fully ruled out considering that exposure variables are aggregated measurements and they may have considerable individual variations. Secondly, the cross-sectional approach prevents us from inferring a causal relationship between exposure and outcome, and only associations can be concluded. Thirdly, the small number of centers included in our multilevel models may have resulted in spuriously low *p*-values. Fourthly, exposure variables were measured in the capital cities of provinces, and there may be certain differences for centers not located in these capitals (for example, Cantabria) or for those that are located in the capital city but for which the measured area of sun exposure does not coincide with the GAN-surveyed areas. Fifthly, self-reporting of asthma prevalence by participants or their parents may produce some information bias. Sixthly, potential confounding factors such as air pollution, lifestyle, food or food supplement intake, and indoor environmental and social determinants were not included in the analysis [64,65,66]. Finally, some residual confounding could remain; we also used the means of meteorological variables from long time periods.

Nevertheless, the results of the present study suggest a protective effect of sun exposure on the prevalence of asthma in adolescents, with GSSI having a greater effect than SHs. Solar irradiation may have a beneficial effect against several diseases, and increasing research in this area suggests revising the recommendations for sun exposure [26,44,67,68]. However, the harmful effects of sun irradiation should also be taken into account.

Further epidemiological observational studies in different geographical areas would be useful to increase knowledge on the causes of sun-exposure- and climate-related asthma; these results may help improve asthma prevention and control. Additionally, prospective cohort studies beginning during pregnancy and following participants through infancy and childhood would be advantageous to investigate the causal relationship between sun exposure and asthma incidence.

## 5. Conclusions

The results of the present study suggest that sun exposure, measured either by SHs or GSSI, may be protective against asthma in adolescents, while in schoolchildren, the associations between sun exposure and asthma prevalence were not significant, although they followed a similar trend. Confirmation of these findings in other geographical settings would improve our understanding of the role of sun exposure in asthma prevention and control.

## Figures and Tables

**Table 1:** Model comparison and model fitness for the multilevel regression analysis.

Parameters	Null Model	Sunshine Hours Model	Solar Irradiance Model
Estimated parameters (k)	2	8	8
6–7 years			
Asthma ever			
Random effects			
Variance	0.2442 (0.077–0.771)	0.0055 (0.001–0.037)	0.0067 (0.001–0.038)
ICC% ^1^	6.90 (2.30–19.0)	0.17 (0.03–1.08)	0.20 (0.04–1.15)
MOR ^2^	1.60	1.07	1.08
PCV ^3^	1	97.7%	97.3%
Model comparison			
AIC ^4^	12,437.90	12,318.62	12,319.38
BIC ^5^	12,453.37	12,380.47	12,381.24
LL ^6^	−6216.9496	−6161.3075	−6151.893
Deviance	12,433.899	12,322.615	12,303.379
Asthma diagnosis			
Random effects			
Variance	0.2704 (0.086–0.852)	0.0075 (0.001–0.043)	0.0091 (0.002–0.047)
ICC%	7.60 (2.54–20.59)	0.23 (0.04–1.29)	0.28 (0.05–1.40)
MOR	1.64	1.09	1.10
PCV	1	97.2%	96.6%
Model comparison			
AIC	11,375.18	11,258.83	11,260.56
BIC	11,390.67	11,321.76	11,322.49
LL	−5685.5912	−5621.9166	−5622.2819
Deviance	11,371.182	11,242.833	11,244.564
13–14 years			
Asthma ever			
Variance	0.0825 (0.026–0.263)	0.0069 (0.002–0.02)	0.0076 (0.002–0.032)
ICC%	2.45 (0.78–7.39)	0.21 (0.05–0.90)	0.23 (0.05–0.96)
MOR	1.32	1.08	1.09
PCV	1	91.6%	90.8%
Model comparison			
AIC	20,111.95	19,603.04	19,603.45
BIC	20,127.7	19,665.81	19,666.22
LL	−10,053.983	−9793.5189	−9793.7253
Deviance	20,107.966	19,587.038	19,587.451
Asthma diagnosis			
Random effects			
Variance	0.1160 (0.037–0.367)	0.0069 (0.002–0.031)	0.0077 (0.002–0.034)
ICC%	3.41 (1.10–10.0)	0.21 (0.04–0.95)	0.23 (0.05–1.02)
MOR	1.38	1.08	1.09
PCV	1	94.1%	93.4%
Model comparison			
AIC	18,203.27	17,731.19	17,731.65
BIC	18,219.03	17,794.05	17,794.51
LL	−9099.6345	−8857.5938	−8857.8258
Deviance	18,199.269	17,715.188	17,715.65

^1^ ICC: intra-class correlation coefficient. ^2^ MOR: median odds ratio. ^3^ PCV: proportional change in variance. ^4^ AIC: Akaike’s information criterion. ^5^ BIC: Bayesian information criterion. ^6^ LL: log likelihood.

**Table 2:** Prevalence (%) of “asthma ever” and “asthma diagnosis” in 6–7-year-old schoolchildren and 13–14-year-old adolescents in the six centers. Prevalence rates compared by logistic regression analysis. Odds ratios (ORs) and 95% confidence intervals (CIs).

6–7 Years					
	Population	Asthma Ever	Asthma Diagnosis	Asthma Ever	Asthma Diagnosis
		N (%)	N (%)	Crude OR (95% CI)	Crude OR (95% CI)
Bilbao	2707	615 (22.7)	568 (21.0)	4.34 (3.59–5.24)	4.49 (3.68–5.47)
Cantabria	2841	490 (17.2)	429 (15.1)	3.07 (2.54–3.73)	3.01 (2.46–3.67)
Cartagena	3509	362 (10.3)	297 (8.5)	1.70 (1.39–2.07)	1.56 (1.31–1.93)
A Coruña	3407	332 (9.7)	278 (8.2)	1.59 (1.30–1.95)	1.50 (1.21–1.86)
Salamanca	2388	191 (8.0)	166 (7.0)	1.28 (1.03–1.60)	1.26 (1.00–1.59)
Pamplona	2363	150 (6.3)	132 (5.6)	1.00	1.00
Trend				*p*-value = 0.000	*p*-value = 0.000
Total	17,215	2140 (12.4)	1870 (10.9)		
**13–14 years**					
		**Asthma Ever**	**Asthma Diagnosis**	**Asthma Ever**	**Asthma Diagnosis**
		**N (%)**	**N (%)**	**Crude OR (95% CI)**	**Crude OR (95% CI)**
Bilbao	3379	1010 (29.9)	909 (26.9)	2.43 (2.16–2.74)	2.78 (2.45–3.15)
Cantabria	4382	1033 (23.6)	903 (20.6)	1.76 (1.56–1.98)	1.96 (1.73–2.22)
A Coruña	3462	712 (20.6)	595 (17.2)	1.48 (1.30–1.67)	1.57 (1.37–1.79)
Salamanca	3485	665 (19.1)	489 (14.0)	1.34 (1.19–1.52)	1.23 (1.07–1.42)
Pamplona	1798	323 (18.0)	255 (14.2)	1.25 (1.08–1.44)	1.25 (1.05–1.48)
Cartagena	3437	513 (14.9)	402 (11.7)	1.00	1.00
Trend				*p*-value = 0.000	*p*-value = 0.000
Total	19,943	4256 (21.3)	3553 (17.8)		

**Table 3:** Sunshine, global solar surface irradiance, temperature, relative humidity, and per capita gross domestic product in the six centers.

Center	Sunshine (Hours/Year ^1^)	Global Solar Surface Irradiance ^2^ (kWh m^−2^ day^−1^)	Temperature ^1^ (°C)	Relative Humidity ^1^ (%)	GDP ^3^ Per Capita (€)
Bilbao	1610	3.54	14.7	70	30,657
Cantabria	1649 ^4^	3.66 ^4^	14.5	74	23,332
A Coruña	1939	3.86	13.8	75	23,800
Salamanca	2667	4.72	12.2	65	20,421
Pamplona	2240	4.04	12.9	67	30,878
Cartagena	2621	5.13 ^5^	17.6	71	21,236

^1^ Agencia Estatal Meteorología: datos climatológicos 1981–2010 annual mean. ^2^ Sancho-Avila JM et al. Agencia Estatal Meteorología: Atlas radiación solar 1983–2005 daily mean. ^3^ GDP = gross domestic product per capita: mean for the 2016–2019 period, Instituto Nacional Estadística. ^4^ Data for Santander capital city. ^5^ Data for Murcia capital city.

**Table 4:** Prevalence of “asthma ever” and sun exposure: sunshine hours and global solar surface irradiance. Multilevel logistic regression models. Crude and adjusted odds ratios (ORs) and 95% confidence intervals (CIs).

6–7 Year Olds		
Asthma Ever		
	Crude OR (95% CI)	Adjusted ^1^ OR (95% CI)
Sunshine hours ^2^	0.91 (0.86–0.97)	0.82 (0.79–0.85)
Solar irradiance ^3^	0.62 (0.35–1.09)	0.15 (0.10–0.23)
**13–14 Year Olds**		
**Asthma Ever**		
	**Crude OR (95% CI)**	**Adjusted ^1^ OR (95% CI)**
Sunshine hours ^2^	0.94 (0.92–0.97)	0.90 (0.87–0.94)
Solar irradiance ^3^	0.66 (0.53–0.83)	0.37 (0.25–0.54)

^1^ Adjusted for age, sex, temperature, relative humidity, and gross domestic product per capita. ^2^ Mean annual increase of one-hundred hours. ^3^ One kWh m^−2^ day^−1^.

**Table 5:** Prevalence of “asthma diagnosis” and sun exposure: sunshine hours and global solar surface irradiance. Multilevel logistic regression models. Crude and adjusted odds ratios (ORs) and 95% confidence intervals (CIs).

6–7 Year Olds		
Asthma Diagnosis		
	Crude OR (95% CI)	Adjusted ^1^ OR (95% CI)
Sunshine hours ^2^	0.91 (0.85–0.97)	0.81 (0.77–0.84)
Solar irradiance ^3^	0.59 (0.33–1.08)	0.13 (0.08–0.20)
**13–14 Year Olds**		
**Asthma Diagnosis**		
	**Crude OR (95% CI)**	**Adjusted ^1^ OR (95% CI)**
Sunshine hours ^2^	0.93 (0.90–0.96)	0.88 (0.85–0.92)
Solar irradiance ^3^	0.60 (0.47–0.77)	0.32 (0.22–0.47)

^1^ Adjusted for age, sex, temperature, relative humidity, and gross domestic product per capita. ^2^ Mean annual increase of one-hundred hours. ^3^ One kWh m^−2^ day^−1^.

**Table 6:** Permutation test results for the multilevel logistic regression models of crude sunshine hours (SHs) and global solar surface irradiance (GSSI) in relation to “asthma ever” and “physician-diagnosed asthma”. *p*-values and 95% confidence intervals (CIs).

6–7 Year Olds		
	Asthma Ever	Asthma Diagnosis
	*p*-value (95% CI)	*p*-value (95% CI)
SHs	0.08 (0.035–0.152)	0.07 (0.029–0.139)
GSSI	0.25 (0.040–0.347)	0.13 (0.070–0.212)
**13–14 Year Olds**		
	**Asthma Ever**	**Asthma Diagnosis**
	***p*-value (95% CI)**	***p*-value (95% CI)**
SHs	0.02 (0.002–0.070)	0.04 (0.011–0.099)
GSSI	0.01 (0.003–0.054)	0.02 (0.002–0.070)

**Table 7:** Multilevel logistic regression models of crude sunshine hours (SHs) and global solar surface irradiance (GSSI) and “asthma ever” and “asthma diagnosis” with the step-by-step removal of one center in each analysis. Crude odds ratios (ORs) and 95% confidence intervals (CIs).

6–7 Year Olds					
		Asthma Ever		Asthma Diagnosis	
Removed Center	Sun Exposure	OR (95% CI)	*p*-Value	OR (95% CI)	*p*-Value
Bilbao	SHs	0.94 (0.94–1.00)	0.061	0.94 (0.88–1.00)	0.056
	GSSI	0.80 (0.46–1.37)	0.409	0.78 (0.45–1.35)	0.378
Cantabria	SHs	0.91 (0.86–0.99)	0.029	0.91 (0.84–0.99)	0.026
	GSSI	0.67 (0.35–1.29)	0.232	0.64 (0.33–1.28)	0.207
Cartagena	SHs	0.89 (0.83–0.95)	0.001	0.88 (0.83–0.96)	0.001
	GSSI	0.38 (0.18–0.83)	0.015	0.37 (0.16–0.86)	0.021
A Coruña	SHs	0.90 (0.86–0.92)	0.001	0.90 (0.85–0.96)	0.001
	GSSI	0.58 (0.32–1.05)	0.074	0.55 (0.30–1.02)	0.059
Salamanca	SHs	0.91 (0.84–0.95)	0.018	0.90 (0.83–0.98)	0.013
	GSSI	0.65 (0.33–1.29)	0.222	0.62 (0.30–1.27)	0.189
Pamplona	SHs	0.92 (0.88–0.97)	0.001	0.91 (0.87–0.97)	0.001
	GSSI	0.58 (0.38–0.91)	0.018	0.57 (0.36–0.90)	0.015
**13–14 Year Olds**					
		**Asthma Ever**		**Asthma Diagnosis**	
**Removed Center**	**Sun Exposure**	**OR (95% CI)**	***p*-Value**	**OR (95% CI)**	***p*-Value**
Bilbao	SHs	0.96 (0.94–0.98)	0.001	0.95 (0.93–0.97)	0.000
	GSSI	0.75 (0.64–0.87)	0.000	0.69 (0.58–0.81)	0.000
Cantabria	SHs	0.93 (0.90–0.97)	0.000	0.92 (0.89–0.95)	0.000
	GSSI	0.65 (0.50–0.86)	0.002	0.60 (0.45–0.86)	0.001
A Coruña	SHs	0.94 (0.91–0.97)	0.000	0.93 (0.90–0.96)	0.000
	GSSI	0.64 (0.51–0.82)	0.000	0.58 (0.45–0.75)	0.000
Salamanca	SHs	0.92 (0.90–0.95)	0.000	0.91 (0.89–0.94)	0.000
	GSSI	0.62 (0.48–0.81)	0.000	0.58 (0.43–0.78)	0.000
Pamplona	SHs	0.95 (0.91–0.98)	0.000	0.93 (0.90–0.96)	0.000
	GSSI	0.65 (0.54–0.79)	0.000	0.59 (0.48–0.72)	0.000
Cartagena	SHs	0.95 (0.92–0.99)	0.007	0.93 (0.90–0.97)	0.000
	GSSI	0.65 (0.45–0.96)	0.030	0.55 (0.37–0.82)	0.003

## Data Availability

The GAN Phase I data from all participating centers, also from Spain, including de-identified individual participant data, will be made available on the Global Asthma Network website, http://www.globalasthmanetwork.org/, within 12 months of all GAN Phase I analyses being published. Access will require a formal request, a written proposal and a signed data access agreement.
